# Enhancing precision in percutaneous vertebroplasty: a study on patient-specific 3D-printed guides for osteoporotic vertebral compression fractures

**DOI:** 10.3389/fsurg.2026.1800908

**Published:** 2026-05-28

**Authors:** Jia-Wen Liang, Li-Na Qiao, Ying-Ao Jia, Liang Xue, Ming-Yang Zhao, Fei Wang

**Affiliations:** 1 Department of Orthopaedics, Affiliated Hospital of Yan'an University, Yan'an, Shaanxi Province, China; 2 Department of Pathology, Affiliated Hospital of Yan'an University, Yan'an, Shaanxi Province, China

**Keywords:** 3D-printed, computer-Aided design, osteoporosis, percutaneous vertebroplasty, vertebral compression fracture

## Abstract

**Objectives:**

The aim of this study was to investigate the safety and feasibility of 3D-printed puncture guides assisting in unilateral-approach percutaneous vertebroplasty (PVP) for the treatment of osteoporotic vertebral compression fractures (OVCFs).

**Methods:**

We retrospectively analyzed the medical records of 106 patients admitted to the Department of Spine Surgery of the Affiliated Hospital of Yan'an University who underwent PVP with a unilateral approach from June 2020 to June 2022, and divided them into 2 groups based on the use of 3D-printed perforation guides during the operation: 52 patients in the 3D-printed perforation guide-assisted PVP group (Observation group), and 54 patients in the traditional PVP group (Control group). The general information, operation time, puncture time, number of intraoperative C-arm fluoroscopies, amount of bone cement injected, postoperative complications, pedicle wall breaches by the puncture needle trajectory, and the degree of compression of the anterior margin of the vertebral body and the Cobb's angle, which were measured on the lateral views of the injured vertebrae preoperatively and postoperatively, were recorded and compared between the two groups. A visual analogue scale (VAS) was used to evaluate the degree of pain in the injured vertebral region, and the Oswestry Disability Index (ODI) was used to assess the degree of functional disability of the patients.

**Results:**

(1) There were no statistically significant differences between the two groups regarding gender, age, body mass index (BMI), bone mineral density (BMD), injured segment, and other baseline characteristics (*P* > 0.05). (2) The operation time, intraoperative puncture time, and cement leakage rate in the observation group were lower than those in the control group (*P* < 0.05). Furthermore, the incidence of nerve root injury, whole-cement displacement, contralateral diffusion, and puncture paths breaching the pedicle walls was significantly lower in the observation group compared to the control group (*P* < 0.05). (3) The degree of vertebral anterior compression in the observation group at 1 day postoperatively [(21.01 ± 1.98)%] was significantly lower than that in the control group [(23.21 ± 2.47)%] (*P* < 0.05). (4) There were no statistically significant differences in the amount of bone cement injected or the incidence of adjacent vertebral fractures between the two groups (*P* > 0.05). (5) There was no statistically significant difference in the comparison of the VAS scores and ODI scores of the two groups in the postoperative period and during the postoperative follow-up period (*P* > 0.05).

**Conclusion:**

3D-printed puncture guide-assisted unilateral approach PVP for the treatment of single-segment OVCFs has significant clinical advantages compared with the traditional unarmed puncture group. Specifically, it improves the ratio of overall to contralateral bone cement dispersion volume, significantly enhances puncture accuracy, and reduces the incidence of surgical complications. It also reduces the number of fluoroscopies, shortens the operative time, and comprehensively improves surgical safety and operational efficiency.

## 1 Introduction

Osteoporotic vertebral compression fracture is a common spinal disorder; following the fracture, patients experience significant pain and severe functional limitations, which seriously impact their health status, quality of life, psychological well-being, and economic burden. This susceptibility is primarily attributed to compromised bone strength and toughness in patients with osteoporosis, rendering the spine (particularly at the thoracolumbar junction) more prone to fracture (1). Nearly half of the OVCFs in the thoracolumbar spine are unstable fractures, which can cause severe neurologic dysfunction, deformity, and disability. With the increasing incidence of fracture sparing and the progressively earlier age of onset, OVCFs has become one of the serious complications of osteoporosis (2, 3). Although conservative treatment can heal cases where vertebral deformity is mild and the anterior vertebral height decreases by less than one-fourth, this approach carries a risk of vertebral deformity, spinal instability, limited mobility, and chronic low back pain. Furthermore, prolonged bed rest associated with such treatment may lead to complications such as bedsores and deep vein thrombosis in the lower limbs (4, 5). As a mainstream surgical technique for minimally invasive treatment of OVCFs, PVP has multiple advantages: immediate pain relief, only local anesthesia is needed, short recovery period, convenient operation, high safety, etc., and is in line with the concept of minimally invasive spinal surgery (6). However, traditional freehand PVP relies heavily on the operator's experience, preoperative and intraoperative image interpretation, and intraoperative fluoroscopy. This often results in issues such as repeated C-arm positioning, puncture injuries, cement leakage, and suboptimal cement distribution, potentially compromising surgical outcomes and leading to severe postoperative complications (7).

Although PVP is an efficient and cost-effective treatment modality, freehand puncture is associated with a high level of uncertainty, including insufficient positioning accuracy, the limited tolerance of elderly patients for the surgical position, and procedure-related risks (8). Driven by the rapid maturation and expanding clinical application of 3D printing technology, it has become increasingly prevalent in some orthopedic surgeries. The independently designed puncture guide combined with 3D printing technology can enable surgeons to achieve fast and accurate placement of puncture needles intraoperatively through precise navigation and positioning, minimize both the number of puncture attempts and the radiation exposure for surgeons and patients, mitigate the risk of intraoperative and postoperative complications, and improve the precision and safety of PVP treatment for OVCFs, simultaneously serving as a valuable training tool for novice surgeons mastering PVP techniques for OVCFs (9).

In this study, we analyzed the imaging data processed using Mimics software, clinical outcomes, and surgical complications to investigate the safety and feasibility of 3D-printed puncture guide-assisted PVP in the treatment of osteoporotic compression fractures. By comparing the clinical efficacy of 3D-printed perforator-guide-assisted PVP with that of traditional unarmed PVP, this study provides more therapeutic options for clinicians and offers a practical basis for the further improvement of 3D-printed perforator-guide-assisted surgical techniques.

## 2 Objects and methods

### 2.1 Design

For the retrospective case analysis, two independent *t*-tests were used to compare normally distributed continuous variables between groups. The chi-square test or Fisher's exact test was used to compare categorical data between the two groups. Given the retrospective nature of this study, blinding of the operating surgeons and patients was not possible. To reduce observer bias, all imaging measurements—including the degree of anterior vertebral compression, Cobb angle, total and contralateral bone cement diffusion volume ratios, and pedicle wall breach assessment—were initially recorded by a spine surgery fellow and subsequently verified by a senior spine surgeon who was not the primary operator. Postoperative clinical scores (VAS and ODI) were evaluated by an independent nurse specialist blinded to the group allocation. When the initial evaluation and the verified record were inconsistent, the final data point was determined through consensus review with a third senior surgeon. Because all recorded values in the database represent the final consensus rather than two fully independent repeated measurements, formal inter-observer reliability indices such as intra-class correlation coefficients were not calculated.

### 2.2 Time and place

The trial was conducted from June 2020 to June 2022 at the Department of Spine Surgery, Affiliated Hospital of Yan'an University.

### 2.3 Objects

The medical records of 106 patients with unilateral, single-vertebral OVCF who underwent PVP and were admitted to the spine surgery ward of the Affiliated Hospital of Yan'an University from June 2020 to June 2022 were retrospectively reviewed.

**Inclusion criteria:** (1) after imaging, the diagnosis of OVCFs was confirmed, (2) the indications for PVP treatment were met, (3) dual-energy x-ray bone densitometry *T*-value ≤−2.5, (4) unilateral, single vertebral body puncture, (5) age ≥55 years old, (6) fracture time <2 weeks, (7) written informed consent obtained from the patient and their family, (8) follow-up duration ≥ 12 months with complete medical records.

**Exclusion criteria:** (1) non-OVCFs and other types of fractures, (2) multi-segmental thoracolumbar vertebral fractures, (3) vertebral body height compression ≥75%, (4) fracture involving the posterior wall of the vertebral body and neurological dysfunction, (5) burst fracture, (6) severe bleeding tendency or coagulation dysfunction, (7) cardiac, pulmonary, renal dysfunction, (8) endocrine, immune system diseases or history of malignant tumors.

Medical records of 122 patients with OVCFs who received unilateral single-segment PVP at the Department of Spine Surgery, Affiliated Hospital of Yan'an University, were initially screened from June 2020 to June 2022. Among them, 16 patients were excluded according to the pre-specified inclusion and exclusion criteria, including 4 cases of multi-segmental thoracolumbar fractures, 3 with vertebral body height compression ≥75%, 2 with burst fractures, and 7 with incomplete follow-up data (duration <12 months). The overall follow-up dropout rate of this study was 5.74% (7/122), and all these patients were excluded from the final analysis as predefined.Finally, 106 eligible patients with OVCFs were enrolled in this study and allocated to two groups based on the surgical technique: 52 patients received 3D-printed puncture guide-assisted PVP (observation group), and 54 patients underwent traditional freehand PVP (control group). The baseline characteristics including gender, age, BMI, BMD and injured vertebral segment were comparable between the two groups, with no statistically significant differences (*P* > 0.05). Specifically, the observation group had 11 males and 41 females with a mean age of (69.35 ± 9.38) years, and the fractured levels included T6 (*n* = 4), T7 (*n* = 1), T8 (*n* = 2), T9 (*n* = 1), T11 (*n* = 5), T12 (*n* = 8), L1 (*n* = 12), L2 (*n* = 9), L3 (*n* = 3), L4 (*n* = 6), and L5 (*n* = 1). The control group had 15 males and 39 females with a mean age of (70.96 ± 10.78) years, and the fractured levels included T7 (*n* = 2), T8 (*n* = 5), T9 (*n* = 1), T11 (*n* = 5), T12 (*n* = 9), L1 (*n* = 16), L2 (*n* = 4), L3 (*n* = 6), L4 (*n* = 5), and L5 (*n* = 1).

### 2.4 Objects

#### 2.4.1 Preoperative preparation

**Figure 1:**
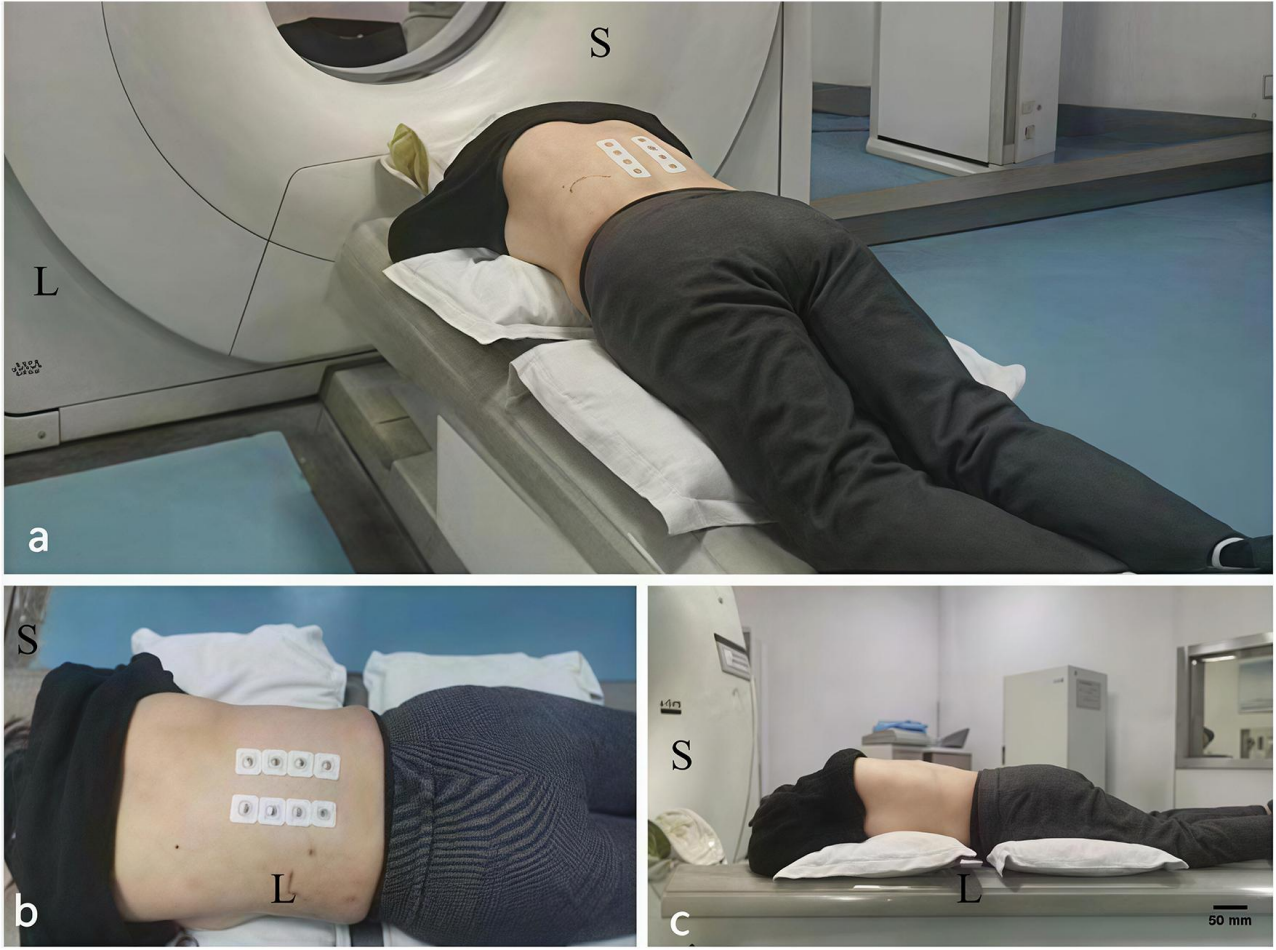
Preoperative CT data acquisition and standardized patient positioning for 3D-printed guide design. **(a)** Lateral view of the patient in prone position for CT scanning; **(b)** dorsal view of the patient with fixed positioning electrode pads; **(c)** lateral view of the standard intraoperative prone position. Orientation: S = Superior, L = Left. Scale bar = 50 mm for the whole figure, calibrated by the standard 33 mm-side-length positioning electrode pads in the images.

In this study, both groups of patients underwent routine preoperative laboratory tests after admission, and specialized evaluations were conducted using thoracolumbar spine x-ray, CT, MRI, and bone density testing to comprehensively assess general physical condition and ensure that preoperative criteria were met. Preoperative CT data were collected, with the patient positioned prone during the preoperative CT examination, matching the intraoperative position. Positioning electrode sheets were placed to encompass the injured vertebrae and adjacent levels, and the area was marked with a surgical marker. The scope of the CT scanning included all the marked points, and the image data were saved (Figure 1).

The data were imported into the medical image processing software Mimics 21.0, where the bone tissue of the injured vertebrae was extracted using Thresholding in Mimics. A mask was generated and then manually corrected to remove unwanted soft tissue and artifacts. A three-dimensional STL model was generated using the Calculate 3D option. The model was processed using the “Smoothing” tool to refine the surface of the spinal model. Edit Mask was used to correct artifacts and noise. The STL file was exported to 3-Matic for editing. A suitable area on the vertebral body surface was selected as the fitting surface of the guide plate. Boolean operations were used to make the base of the guide plate closely fit the bone surface, and a 2–3 mm thickness was created for the base using the Offset tool to ensure sufficient strength and stability. Based on preoperative imaging, the ideal puncture entry point and needle angle were determined based on the surgeon's experience. A target point was designed by analyzing the height, morphology, and fracture lines of the injured vertebral margins. The pedicle with the larger diameter on the injured side was selected as the puncture trajectory. The puncture path was defined as the line connecting the outer edge of the injured vertebral body to the base of the transverse process; the intersection of this line with the dorsal skin surface defined the entry point, while the angle between this extension and the guide plate defined the puncture angle. The location and orientation of the puncture channel were determined in 3-Matic software, and the Cylinder Tool was used to create a puncture hole (2.5 mm or 3 mm in diameter) in the guide plate. The puncture holes were oriented according to preoperative planning, usually with a slight upward tilt to allow precise access to the vertebral body. The length of the puncture channel was optimized to avoid disturbing the surrounding bone structure, and the edges were smoothed to prevent intraoperative slippage or interference with fluoroscopy. ”A latticed support structure was then generated using” Lattice “to reduce the weight of the guide plate.” A Hollow “treatment was applied to hollow out the inside of the guide plate, reducing material usage while maintaining strength. Mesh Repair was performed in 3-Matic to check for holes or non-manifold surfaces. Thickness Analysis was used to ensure that the guides possessed sufficient strength after 3D printing. The “Measurements” tool was used to verify whether the angle and position of the puncture holes were consistent with the preset scheme. Finally, the digital model of the designed 3D-printed puncture guide was exported in STL format and loaded onto a Raise3D Pro2 3D printer (Raise3D Inc., Shanghai, China) for fabrication. Medical-grade polylactic acid (PLA) filament (1.75 mm diameter) was selected as the printing material for its excellent biocompatibility and mechanical stability, making it suitable for clinical intraoperative applications. The printer utilizes fused filament fabrication (FFF) technology with a standard 0.4 mm diameter nozzle, and the printing parameters were set as follows: layer thickness of 0.1 mm, nozzle temperature of 210 °C, heating platform temperature of 60 °C, printing speed of 60 mm/s, 100% infill density, *XY*-axis positioning accuracy of 0.78125 μm, and *Z*-axis positioning accuracy of 0.078125 μm.

**Figure 2:**
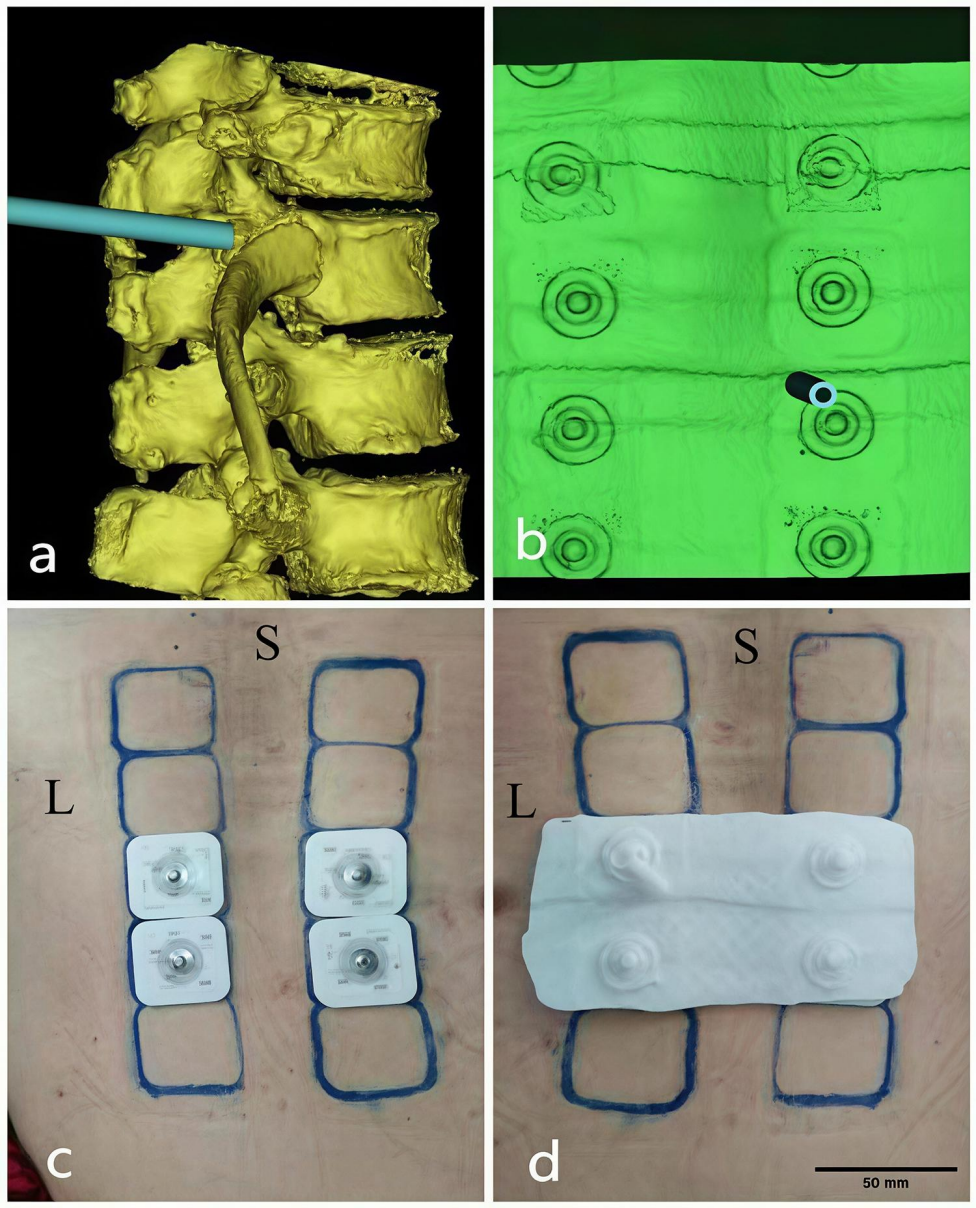
Design and preoperative verification of the customized 3D-printed puncture guide. **(a)** Preoperative puncture trajectory planning on the 3D reconstructed vertebral model; **(b)** 3D reconstructed skin surface model with positioning marks; **(c)** Preoperative body surface positioning with electrode pads; **(d)** Preoperative fitting verification of the 3D-printed guide. Orientation: S = Superior, L = Left. Scale bar = 50 mm for the whole figure, calibrated by the standard 33 mm-side-length positioning electrode pads in the images.

After printing, the support structure was completely removed, and the surface of the guide was polished to remove burrs and sharp edges. The fit of the guide base to the preoperative 3D vertebral model, the patency of the puncture channel, and the consistency of the puncture angle with the preoperative design were individually verified. Qualified guides were transferred to the hospital sterile supply center for ethylene oxide (EtO) low-temperature sterilization (sterilization cycle: 12 h, aeration time: 7 d), followed by aseptic packaging and sealed storage before intraoperative use (Figure 2).

#### 2.4.2 Surgical method

**Figure 3:**
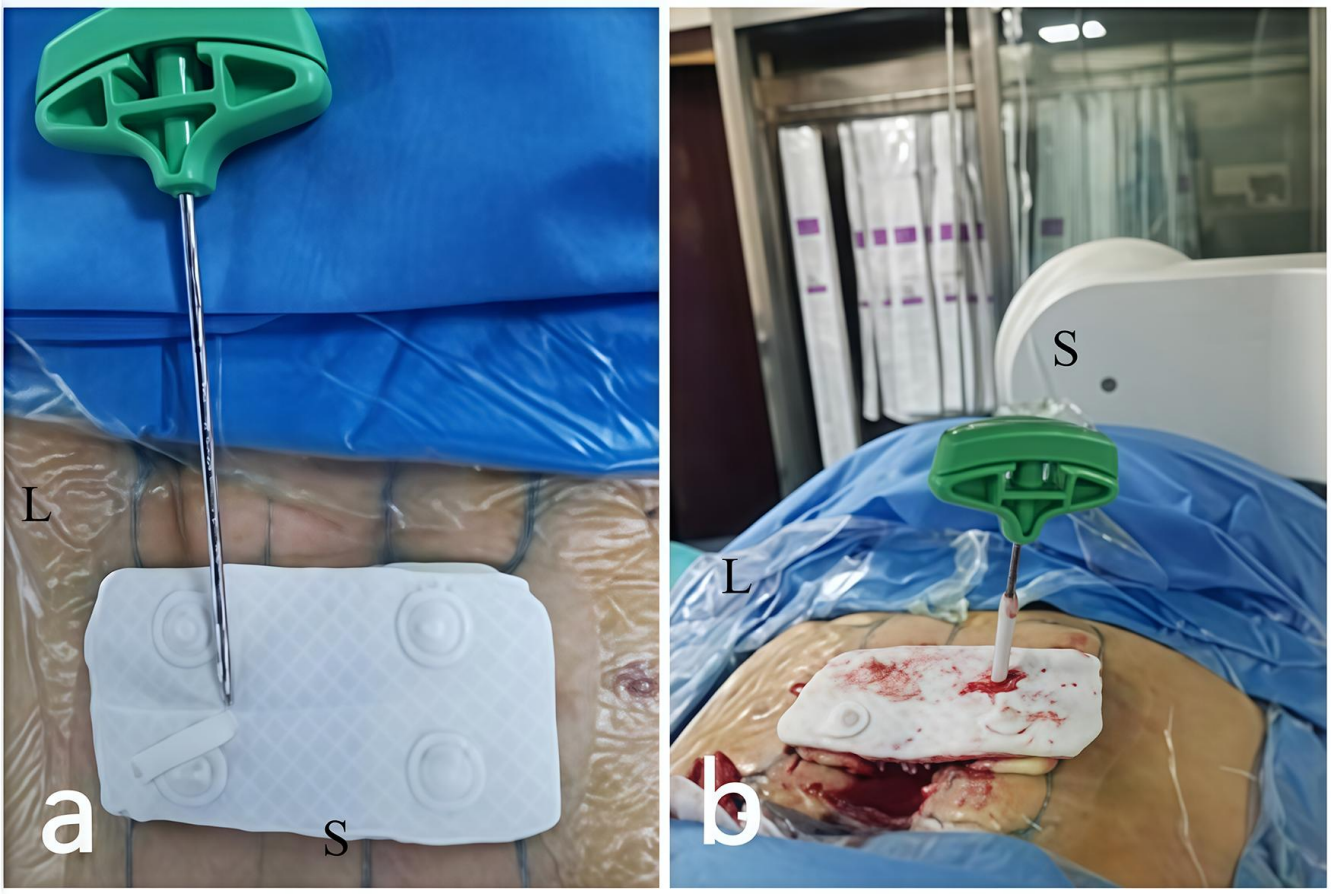
Intraoperative puncture procedure assisted by the 3D-printed guide. **(a)** Puncture needle inserted along the preset channel of the guide; **(b)** Puncture needle placed at the pre-designed target position. Orientation: S = Superior, L = Left. Scale calibrated by the standard 33 mm-side-length electrode pads and standard puncture needle in the images.

All procedures were performed by the same group of senior surgeons, and the same brand, batch and specification of bone cement were used in both groups to ensure operational consistency and eliminate confounding factors. The OSTEOPAL®V polymethylmethacrylate (PMMA) bone cement (Heraeus Medical GmbH, Wehrheim, Germany; medical device registration number in China: Guoxie Zhujin 20223130880) used in this study was prepared strictly according to the product instruction manual revised in December 2024. At a room temperature of 25 °C, the polymerization process of the bone cement is divided into four phases: mixing phase (0–1 min), waiting phase (1–3 min), application phase (3–9 min), and curing phase (9–18 min). The bone cement was loaded into a dedicated PVP syringe immediately after mixing into a uniform paste at the end of the waiting phase (early application phase, ∼3 min after mixing), when the viscosity was low enough for smooth and controllable injection. The bone cement was injected slowly and intermittently under real-time lateral C-arm fluoroscopic guidance. The injection was paused for approximately 3–5 s after each small bolus to observe cement distribution. If paravertebral, perivascular, or intervertebral cement leakage was observed, the injection was stopped immediately and resumed only after the cement viscosity increased appropriately. All injections were completed within the application phase (within 9 min after mixing). The injection was terminated once satisfactory bilateral cement dispersion across the vertebral midline was confirmed, or when the cement reached the posterior quarter of the vertebral body to avoid posterior leakage. Procedures in the observation group were performed using a 3D-printed puncture guide. The specific procedure was as follows: the patient was positioned in the standard prone position on the operating table with the positioning patch. The guide plate was sterilized at low temperature beforehand. After routine sterilization of the surgical field and sterile draping to establish a sterile surgical area, the channel hole of the sterile guide plate was aligned precisely with the positioning patch and stabilized manually. Then local infiltration anesthesia was administered according to the guide plate positioning. After the anesthesia took effect, a small transverse incision of approximately 5 mm was made in the skin. The puncture guide was then stabilized manually, and the needle was inserted through the guide channel under C-arm fluoroscopic guidance to slowly advance to the surface of the pedicle. The C-arm was adjusted by the off-stage physician to the standard anterolateral view. Once fluoroscopy confirmed no abnormalities, the puncture needle was then inserted into the bone along the guide plate channel and advanced further to the ideal position (anterior 1/3–1/4 of the vertebral body) (Figure 3). The bone cement was mixed to the appropriate consistency. A small amount was injected slowly under x-ray fluoroscopic guidance. The injection was paused for approximately 3–5 s to observe cement distribution and was stopped once satisfactory dispersion was confirmed.

In the control group, the traditional freehand puncture method was used for the operation. The procedure involved adjusting the patient to the standard prone position, administering local infiltration anesthesia approximately 2–3 cm lateral to the spinous process at the level of the injured vertebral arch root, and making a 5-mm transverse incision; subsequently, the needle angle was adjusted under repeated x-ray fluoroscopy, guiding the needles to the target position, defined as follows: in the anteroposterior view, the needle tip must not breach the medial or lateral walls of the pedicle, while in the lateral view, the tip should reach the posterior wall of the vertebral body before being advanced to the final destination (anterior 1/3–1/4 of the vertebral body). Bone cement was prepared to the appropriate consistency, and a small quantity was injected slowly under x-ray fluoroscopic guidance. Injection was paused for approximately 3–5 s to verify cement distribution, and the procedure was halted once satisfactory dispersion was achieved.

During the whole process, the physician operated while observing the patient's activities of both lower limbs to prevent nerve injury, and stopped the injection when the ideal filling situation was reached or leakage occurred. Postoperatively, incisions were covered with sterile dressings, and patients were maintained in the prone position for 10–15 min to ensure complete cement curing; they were then repositioned supine and transferred to the ward for continuous vital sign monitoring, with a normal diet resumed if all clinical parameters remained stable within 6 h.

### 2.5 Indicators for evaluating efficacy

**Figure 4:**
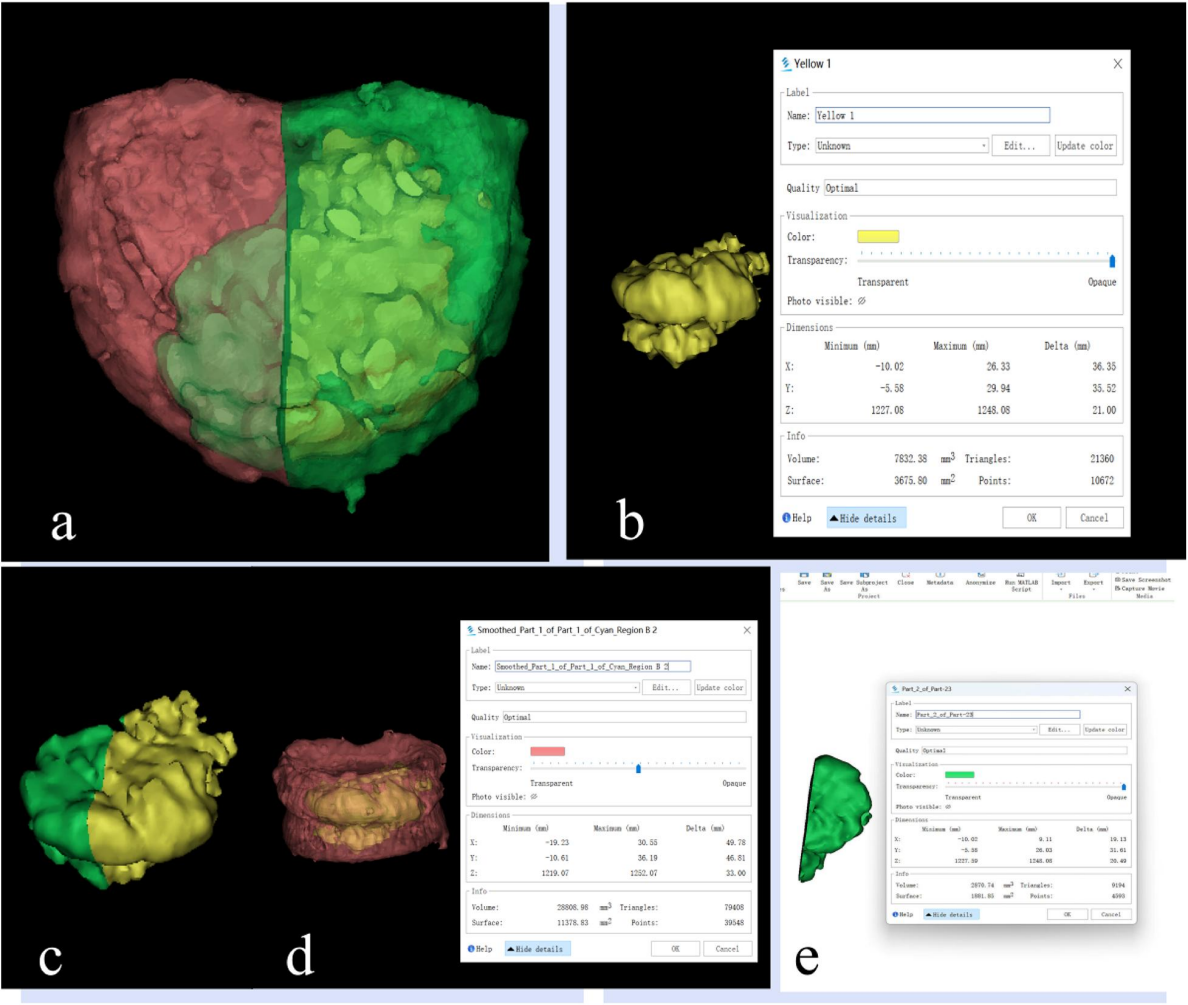
Quantitative measurement of bone cement diffusion volume ratio via mimics 3D reconstruction. **(a)** Vertebral body model divided into puncture side and contralateral side along the sagittal midline; **(b)** segmented bone cement model; **(c)** bone cement model divided by the vertebral midline; **(d)** total vertebral body volume (Vt) measurement; **(e)** contralateral cement volume (Vc) measurement. Contralateral diffusion ratio = Vc/Vt × 100%.

**Figure 5:**
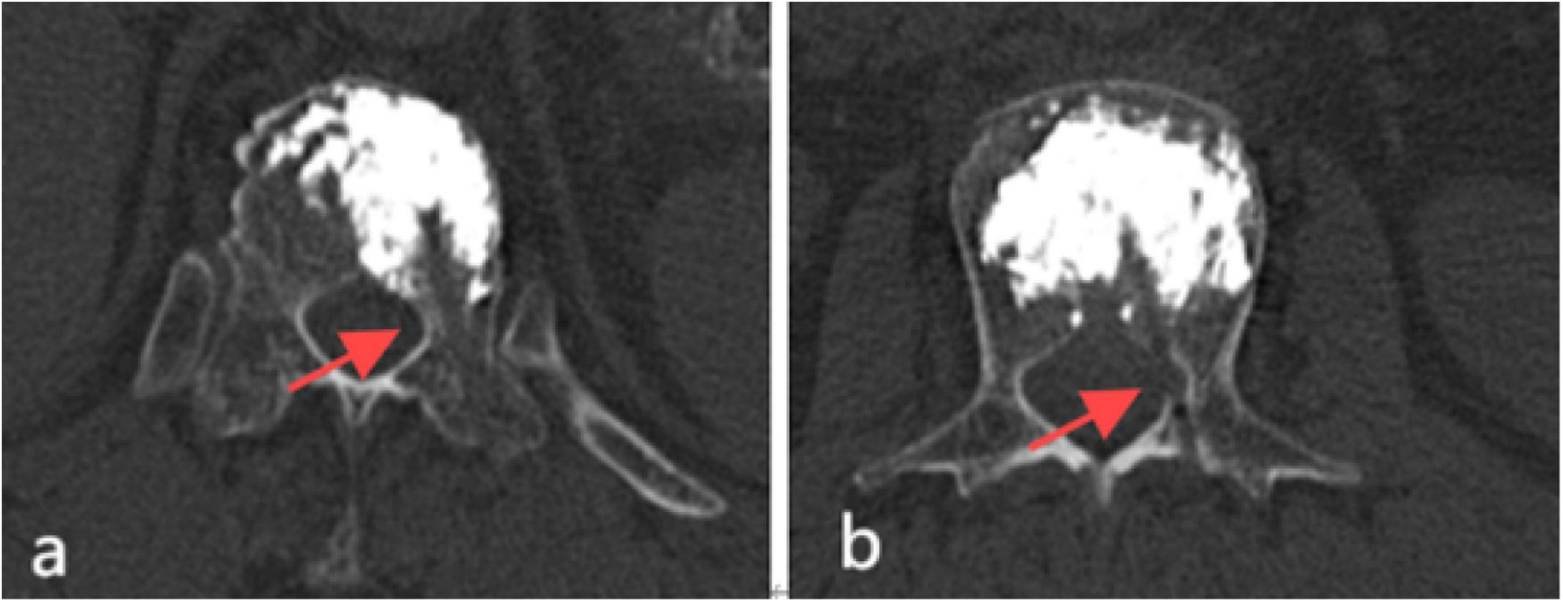
Evaluation of puncture trajectory accuracy via postoperative axial CT. **(a)** Puncture trajectory completely contained within the pedicle cortex; **(b)** puncture trajectory breached the medial pedicle wall (red arrow).

1. The length of surgery, puncture time, number of intraoperative fluoroscopies, degree of compression of the anterior margin of the injured vertebrae (%), and Cobb's angle at 1 d postoperatively, VAS scores, and ODI scores during the postoperative and postoperative follow-up periods were collected and compared between the two groups.
2. CT scans of the operated segments were performed on the postoperative patients, and the CT data were reconstructed in three dimensions using Mimics 21.0 medical image processing software to quantitatively analyze the distribution of the cement in the injured vertebrae, calculate the total volume of the cement in the injured vertebrae (Vt) and the volume of the contralateral distribution (Vc), and then calculate the contralateral distribution ratio, as illustrated in Figure 4.
3. Based on postoperative axial CT images, the puncture needle trajectory was assessed for breaches of the medial and lateral pedicle walls (Figure 5).
4. The incidence of postoperative complications, such as cement leakage and nerve root injury symptoms, was collected and compared between the two groups. Nerve root injury symptoms mainly include new pain, sensory abnormalities (such as numbness, pins and needles sensation, localized hypoesthesia or hyperesthesia), decreased muscle strength, and reflex abnormalities (ankle reflexes, knee reflexes, etc., are weakened or disappeared).

### 2.6 Statistical methods

SPSS 26.0 software was used for statistical analysis of data in this study. Measurements including age, BMD, BMI, operative time, intraoperative puncture time, number of intraoperative fluoroscopies, amount of cement injected, overall diffusion volume ratio of cement, contralateral diffusion volume ratio, degree of compression of anterior vertebral body margin, Cobb's angle, VAS score, and ODI score were first tested for normality using the Kolmogorov–Smirnov test. Data that were normally distributed were expressed as mean ± SD, and comparisons between groups were performed using the independent samples *t*-test. Comparisons of categorical data between the two groups, such as gender, injured vertebral segment, postoperative complications, and whether the puncture path breached the inner and outer walls of the pedicle, were analyzed using the chi-square test or Fisher's exact test. Statistical significance was defined as a *p*-value <0.05. To ensure data accuracy, all imaging and clinical parameters were verified by two independent assessors, and any discrepancies were resolved by discussion with a third senior investigator. Only consensus values were entered into the final database; therefore, no formal intra- or inter-rater reliability coefficients were computed. Sample size was calculated based on the primary endpoint (incidence of bone cement leakage) using a two-sided test for two independent sample rates, with a pre-specified type I error rate *α* = 0.05 and test power 1 − *β* = 80%. The expected cement leakage rate was set as 5.8% in the 3D-printed guide group and 18.5% in the traditional freehand PVP group, according to prior related studies and our center's clinical data. The minimum required sample size of 50 patients per group was verified by PASS 15.0 software. *Post-hoc* statistical power analysis was performed using G*Power 3.1.9.7 software based on the actual enrolled data to confirm the statistical reliability of the study results.

## 3 Results

### 3.1 Comparison of general information

All imaging measurements and clinical scores were recorded through a consensus-based verification process involving at least two observers. No data points required exclusion due to unresolved disagreement. There was no statistically significant difference in the comparison of general information such as gender, age, BMI, BMD, and injured vertebral segment between the two groups of patients (*P* > 0.05) (Table 1).

**Table 1:** Comparison of the general data of the two groups of patients.

Characteristic	Observation group (*n* = 52)	Control group (*n* = 54)	*t/χ*^2^ value	*P* value
Sex (male/female)	11/41	15/39	0.628	0.428
Age (years), mean ± SD	69.35 ± 9.38	70.96 ± 10.78	0.823	0.413
BMD (g/cm^2^), mean ± SD	−4.21 ± 1.23	−4.14 ± 1.30	0.272	0.786
BMI (kg/m^2^), mean ± SD	22.64 ± 3.33	23.28 ± 2.75	1.018	0.283
Injured vertebral segment (*n*)			7.333	0.602
T8	2	5		
T9	1	1		
T11	5	5		
T12	8	9		
L1	12	16		
L2	9	4		
L3	3	6		
Others	12	8		

### 3.2 Sample size and statistical power verification

The final enrolled 52 patients in the observation group and 54 patients in the control group exceeded the pre-calculated minimum sample size (50 per group). *post-hoc* analysis showed a statistical power of 83.2% for the primary endpoint (bone cement leakage incidence), and >99% statistical power for key secondary endpoints including operation time, puncture time, and intraoperative fluoroscopy frequency, confirming sufficient statistical power of this study.

### 3.3 Comparison of surgery-related indicators

Unilateral PVP was successfully performed in all 106 cases of single vertebral fractures. The operative time in the observation group was (44.13 ± 5.34) min, and the operative time in the control group was (51.19 ± 9.20) min; the difference was statistically significant in comparison (*P* < 0.05). The volume of bone cement injected in the observation group was (5.95 ± 1.18) mL, and the amount of bone cement injected in the control group was (5.72 ± 1.26) mL; the difference was not statistically significant in comparison (*P* > 0.05). The number of fluoroscopies in the observation group was (18.85 ± 2.29) times, and the number of fluoroscopies in the control group was (28.24 ± 3.43) times, and the difference was statistically significant (*P* < 0.05). The puncture time was (6.87 ± 1.01) min in the observation group and (10.54 ± 1.04) min in the control group; the difference was statistically significant (*P* < 0.05). The overall diffusion volume ratio of bone cement was (24.10 ± 4.13)% in the observation group and (19.69 ± 4.82)% in the control group. The contralateral diffusion volume ratio of bone cement was (14.64 ± 3.50)% in the observation group and (12.33 ± 3.44)% in the control group; the difference was statistically significant (*P* < 0.05) in comparison (Table 2).

**Table 2:** Comparison of surgery-related indicators between the two groups of patients(mean ± SD).

Variable	Observation group(*n* = 52)	Control group(*n* = 54)	*t* value	*P* value
Operation time (min)	44.13 ± 5.34	51.19 ± 9.20	−4.799	<0.001
Intraoperative puncture time (min)	6.87 ± 1.01	10.54 ± 1.04	−18.420	<0.001
Intraoperative fluoroscopies (times)	18.85 ± 2.29	28.24 ± 3.43	−16.523	<0.001
Bone Cement Injection Volume (mL)	5.95 ± 1.18	5.72 ± 1.26	0.953	0.343
Bone cement diffusion volume ratio (%)				
Total	24.10 ± 4.13	19.69 ± 4.82	5.051	<0.001
Contralateral	14.64 ± 3.50	12.33 ± 3.44	3.425	<0.001

### 3.4 Comparison of imaging data

Cement leakage occurred in 3 cases (5.8%) in the observation group and 10 cases (18.5%) in the control group. The difference between the groups was statistically significant (*P* < 0.05). There were no symptoms of nerve root injury in the observation group; in the control group, there were 6 cases (11.1%), representing a statistically significant difference (*P* < 0.05). Adjacent vertebral body re-fracture occurred in 2 cases (3.9%) in the observation group and 4 cases (7.4%) in the control group. The difference between the groups was not statistically significant (*P* > 0.05) (Table 3).

**Table 3:** Comparison of the occurrence of surgery-related complications between the two groups (n).

Variable	Observation group(*n* = 52)	Control group(*n* = 54)	*χ*^2^ value	*P* value
Bone cement leakage	3	10	4.002	0.045
Intervertebral disk	1	4		
Paravertebral vein	0	1		
Intravertebral	0	3		
Paravertebral tissue	2	2		
Nerve Root Injury Symptoms	0	6		0.027*
Re-fracture of adjacent Vertebrae	2	4		0.679*

* Fisher's exact probability method was used instead because the chi-square test condition was not met (expected frequency < 5).

The degree of compression of the anterior margin of the vertebral body in both groups at 1 day after surgery was significantly improved compared with that before surgery, and the difference was statistically significant (*P* < 0.05). Similarly, the Cobb angle in both groups at 1 day post-surgery improved significantly compared to baseline; however, the inter-group difference was not statistically significant (*P* > 0.05). At the final follow-up, the Cobb angle in both groups had significantly recovered from pre-surgery values but showed some loss of correction compared to immediate post-surgery measurements; however, this difference was not statistically significant (*P* > 0.05). The compression degree of the anterior edge of the vertebral body was (21.01 ± 1.98)% in the observation group and (23.21 ± 2.47)% in the control group at 1 day after the operation, and the difference was statistically significant in comparison (*P* < 0.05) (Table 4).

**Table 4:** Comparison of cobb's angle and degree of compression at the anterior margin of the injured vertebrae(mean ± SD).

Variable	Observation group(*n* = 52)	Control group(*n* = 54)	*t* value	*P* value
Degree of compression of the anterior margin of the vertebral body (%)				
Preoperative	41.85 ± 4.29	42.32 ± 3.01	−0.666	0.507
Postoperative 1 day	21.01 ± 1.98	23.21 ± 2.47	−5.071	<0.001
Cobb's angle (°)				
Preoperative	27.36 ± 3.96	28.26 ± 4.74	−1.052	0.295
Postoperative 1 day	14.38 ± 2.69	14.56 ± 2.01	−0.388	0.699
Postoperative 12 months	15.78 ± 3.01	15.77 ± 2.50	0.014	0.989

### 3.5 Clinical efficacy

In the control group, the needle trajectory breached the inner or outer pedicle walls in 7 cases, whereas no such breaches occurred in the observation group; this difference was statistically significant (*P* < 0.05) (Table 5).

**Table 5:** Comparison of pedicle wall breach by puncture trajectory (n).

Groups	No pedicle breach	Pedicle breach	*P* value
Observation group(*n* = 52)	52	0	0.013*
Control group(*n* = 54)	47	7	

* Fisher's exact probability method was used instead because the chi-square test condition was not met (expected frequency < 5).

VAS scores and ODI scores of patients in both groups showed significant improvement compared with preoperative levels at postoperative and follow-up time points, whereas although VAS scores in the observation group showed greater improvement than in the control group, the difference between the two groups was not statistically significant (*P* > 0.05). There was no statistically significant difference between the ODI scores of the two groups (*P* > 0.05) (Table 6).

**Table 6:** Comparison of clinical outcomes between the two groups of patients (mean ± SD).

Variable	Observation group(*n* = 52)	Control group (*n* = 54)	*t* value	*P* value
VAS score(points)				
Preoperative	6.96 ± 0.84	6.87 ± 0.83	0.564	0.574
Postoperative 1 day	2.81 ± 0.69	2.61 ± 0.56	1.613	0.110
Postoperative 12 months	0.71 ± 0.46	0.81 ± 0.39	−1.250	0.214
ODI (%)				
Preoperative	60.44 ± 4.49	61.94 ± 4.58	−1.704	0.091
Postoperative 1 day	28.92 ± 3.26	27.80 ± 3.59	1.698	0.094
Postoperative 12 months	12.88 ± 3.21	12.37 ± 2.73	0.890	0.376

## 4 Discussion

OVCFs are among the most common complications of osteoporosis, characterized by severe thoracolumbar pain, spinal deformity, significant activity limitations, and potential neurological or spinal cord dysfunction. The thoracolumbar junction (T11–L1) represents a region with a high incidence of these fractures (10). Conservative treatment for OVCFs requires prolonged bed rest, which may precipitate the progression of vertebral deformity, chronic pain, and systemic complications such as deep vein thrombosis and pressure ulcers, consequently compromising patients'quality of life (11). Therefore, minimally invasive techniques such as PVP have attracted much attention because of their ability to effectively restore spinal morphology and their short perioperative period. After long-term clinical practice and continuous exploration, PVP has achieved remarkable clinical results in the treatment of OVCFs (12). This procedure rapidly restores vertebral body strength, achieves stabilization, and reduces pain, demonstrating significant therapeutic advantages in treating osteoporosis-induced OVCFs (13).

However, conventional manual PVP presents significant technical limitations. This procedure relies heavily on the surgeon's clinical experience and requires repeated adjustments to the puncture trajectory using C-arm fluoroscopy intraoperatively. This not only increases radiation exposure for both the medical staff and the patient but also elevates the risk of puncture-related injuries, suboptimal cement distribution, and cement leakage (8). Although conventional unilateral PVP offers the advantages of minimal trauma and brief operative time, limitations in puncture accuracy often result in uneven cement dispersion and asymmetrical distribution on either side of the vertebral body. This leads to eccentric loading of the vertebral body, increasing the long-term risk of vertebral re-collapse, adjacent vertebral fractures and secondary spinal deformity. In contrast, bilateral puncture allows for a more uniform distribution of bone cement; however, it involves longer operative times, higher radiation exposure, and an increased risk of soft tissue and pedicle injury, making it difficult to apply widely in elderly patients with poor tolerance (14, 15). Although emerging techniques such as percutaneous curved-angle kyphoplasty (PCKP) can optimize cement distribution via a single-site puncture, their procedural complexity and lengthy operation times, compounded by the risk of needle fracture during withdrawal, have hindered their broad clinical implementation (16, 17).

Given the above limitations of existing surgical techniques, there is an urgent clinical need for a more accurate, safe and easy-to-operate puncture navigation scheme for unilateral PVP. In this study, we developed a personalized 3D-printed puncture guide, and confirmed its clinical value through a retrospective controlled study. In this study, the degree of anterior vertebral margin compression, as well as VAS and ODI scores in both groups, showed significant improvement at 1 day postoperatively and during follow-up compared to preoperative values, indicating effective pain relief in both groups; however, the observation group demonstrated significantly superior outcomes compared to the control group, with shorter operative times, fewer fluoroscopies, reduced puncture times, and lower rates of nerve root injury and bone cement leakage. Furthermore, Chen et al. (18) and others demonstrated that 3D-printed body surface guide-assisted PVP, featuring precisely designed needle entry points, angles, and safe trajectories, facilitates rapid and accurate puncture; this shortens overall operative time and minimizes collateral tissue injury, thereby reducing early postoperative pain. Li et al. (19) and others, on the other hand, found that personalized 3D-printed guide templates enabled full visualization of the fractured vertebrae and individual surgical plans, significantly improving puncture stability and accuracy. Xu et al. (20) and others, used Mimics software to design 3D models before surgery, which can realize “targeted PVP”, reduce the number of fluoroscopies, significantly reduce the incidence of cement leakage, and realize the visualization of the injured vertebrae, which is more helpful for the treatment of complex OVCFs patients. These findings indicate that integrating 3D modeling software with 3D printing technology enables surgeons to construct detailed anatomical models and formulate precise surgical plans, ultimately reducing operative time, surgical risks, and radiation exposure.

In the vast majority of previous clinical trials, the diffusion volume of bone cement has been used as one of the main efficacy assessment indices for PVP in the treatment of OVCFs. However, there are limitations in this assessment method. Due to the significant inter-individual volume differences between the thoracolumbar vertebrae (the coefficient of variation of vertebral body volume reaches 35%–42%), the absolute volume of bone cement alone cannot objectively reflect the quality of its distribution within the vertebral body. This study used Mimics software to reconstruct postoperative computed tomography data in three dimensions and to calculate the cement dispersion volume ratio quantitatively. This approach not only quantifies cement filling efficiency (by using ratios to eliminate the confounding effect of variations in vertebral volume) but also allows for an objective assessment of the quality of cement distribution. This approach is similar to using “body fat percentage” instead of body weight to assess obesity; it not only ensures cross-case comparability but also predicts the long-term stability of dispersion morphology, thereby providing a scientific basis for the precise control of bone cement injection (21, 22). Postoperative axial CT images were analyzed to determine whether the puncture trajectory breached the inner or outer pedicle walls, thereby verifying if the 3D-printed puncture guide set improved puncture accuracy. In this study, the overall and contralateral diffusion volume ratios of bone cement in the observation group were (24.10 ± 4.13) % and (14.64 ± 3.50) %, respectively. Both values were significantly higher than those in the control group, where the overall ratio was (19.69 ± 4.82) % and the contralateral ratio was (12.33 ± 3.44) % (*P* < 0.05). In the control group, the puncture needle trajectory breached the inner or outer pedicle walls in 7 cases, whereas no such breaches occurred in the observation group; this difference was statistically significant (*P* < 0.05). In the observation group, the overall and contralateral diffusion volume and the trajectory of the puncture needle did not break through the inner and outer walls of the pedicles, which was significantly better than that of the control group, mainly because of the more precise puncture path assisted by the guide plate, and the tip of the needle was located closer to the target position, which made it easier for the cement to diffuse symmetrically to the two sides.

The diffusion pattern of bone cement is considered one of the key factors influencing the long-term efficacy of PVP. Existing studies indicate that a “spongy” or “diffusive” distribution pattern of bone cement—characterised by thorough diffusion and adequate contact with both the superior and inferior endplates—is associated with better clinical outcomes. This distribution pattern is more effective in restoring vertebral strength and reducing the risk of postoperative residual pain and vertebral re-compression (23, 24). In this study, the use of a 3D-printed puncture guide allowed the needle tip to reach the vicinity of the vertebral midline with greater precision, thereby achieving good diffusion of bone cement to the contralateral side of the midline under unilateral puncture conditions. This improvement in the contralateral diffusion volume ratio not only signifies an expansion of the bone cement filling range but also indicates that the fracture area has gained more comprehensive mechanical support. The observation group demonstrated a greater reduction in the compression rate of the anterior vertebral margin on postoperative day 1 (21.01% vs. 23.21%), a finding that was confirmed radiologically. Effective contralateral diffusion facilitates an even distribution of stress on both sides of the vertebral body, thereby mitigating the eccentric loading caused by unilateral cement filling; theoretically, this may reduce the long-term risk of lateral vertebral collapse or secondary scoliosis (23, 25).

In both groups, the Cobb angle improved significantly postoperatively compared with preoperative levels; however, some degree of rebound was observed at the final follow-up, although the difference between the two groups was not statistically significant. This phenomenon is not uncommon in follow-up studies of PVP procedures and suggests that, even after the bone cement has set, the vertebral body may still undergo progressive height loss or an increase in the kyphotic angle. Studies have suggested that the slight postoperative rebound in the Cobb angle may be associated with various factors, including micro-movement at the interface between the bone cement and the surrounding trabecular bone, the continued progression of osteoporosis, and stress concentration effects at the vertebral endplates (26). Finite element analysis has also indicated that bone cement anchored to the superior and inferior endplates can more effectively resist axial loads, thereby reducing the risk of further loss of vertebral height (24, 27). In this study, there was no significant difference in the degree of Cobb angle rebound between the two groups, suggesting that although bone cement diffusion was superior in the observation group, both surgical groups achieved stable spinal alignment overall. However, the clinical significance of this rebound trend warrants attention. Although limited in magnitude, persistent loss of vertebral height remains an important issue that warrants clinical monitoring postoperatively (28, 29). Therefore, standardised postoperative anti-osteoprosis treatment, specifically by improving bone density to enhance the stability of the bone-cement interface, is crucial for mitigating or preventing the progressive increase in the Cobb angle.

This study employed Mimics-based 3D reconstruction quantification to evaluate the efficacy of 3D-printed guide-assisted PVP. Using the total bone cement diffusion volume ratio and the contralateral diffusion volume ratio as core indicators, it addressed the limitations of traditional two-dimensional x-ray measurements, which are susceptible to substantial measurement errors, and established a more objective and precise quantification scheme for bone cement distribution. Building on this, the personalised guide enables sufficient trans-midline cement dispersion via a unilateral approach.While retaining the advantages of unilateral minimally invasive surgery, it achieves vertebral biomechanical stability comparable to that of bilateral uniform dispersion, thereby providing a viable solution to the long-standing clinical dilemma of balancing minimally invasive procedures with optimal cement dispersion. Safety data from 106 cases demonstrated an absence of pedicle wall perforation in the guide plate group, with a significantly lower incidence of cement leakage and nerve root injury, thereby providing evidence-based support for the widespread adoption of this technique.

The limitations of this study warrant objective consideration. Despite controlling for bias through strict inclusion and exclusion criteria, the single-centre retrospective design cannot entirely eliminate inherent biases, and the conclusions require validation through multicentre prospective randomised controlled trials. The inclusion of subjects was limited to single-segment OVCFs with unilateral puncture, excluding cases of multisegmental fractures, severe compression, and burst fractures; while this effectively controlled for confounding factors, it also limited the applicability of the findings to complex cases. From a technical perspective, the guide was designed based on preoperative prone CT scans; changes in patient position during surgery may cause displacement of skin landmarks and affect the accuracy of alignment, with potential for greater deviation in the thoracic segments. Although intraoperative fixation of the electrode pads can partially mitigate this issue, further optimisation is required. Furthermore, as this study did not incorporate finite element analysis, it is unable to elucidate the biomechanical mechanisms by which cement-side diffusion optimisation affects vertebral stress distribution and long-term mechanical stability. The 12-month follow-up period reflects only mid-term outcomes; long-term adverse events such as vertebral re-collapse, secondary scoliosis and adjacent vertebral fractures require observation over a longer period to be clarified.

Future work will focus on the following areas: conducting a multicentre prospective RCT to enhance the level of evidence, utilising finite element analysis to elucidate the biomechanical mechanisms of contralateral diffusion optimisation, optimising guideplate design to minimise the impact of postural changes on alignment accuracy, expanding the indications to include complex OVCFs, and extending the follow-up period to 3–5 years. In the long term, head-to-head comparisons will be initiated with technologies such as robot-assisted PVP and kyphoplasty to clarify the respective advantages of each approach and identify optimal patient populations, thereby providing more precise clinical decision-making criteria for personalised minimally invasive treatment.

## 5 Conclusions

This study demonstrates that unilateral PVP assisted by 3D-printed puncture guides has significant clinical and technical advantages in the treatment of single-segment OVCF, which can effectively improve the accuracy and safety of surgery through precise preoperative planning and simulation. Specifically, this approach enhances puncture precision, reduces iatrogenic injuries and the time required for intraoperative parameter adjustments, thereby improving surgical efficiency, and simplifies the operative workflow while significantly reducing intraoperative radiation exposure to patients and surgical staff.

## Data Availability

The original contributions presented in the study are included in the article/Supplementary Material, further inquiries can be directed to the corresponding author.
